# A novel sweat sensor detects inflammatory differential rhythmicity patterns in inpatients and outpatients with cirrhosis

**DOI:** 10.1038/s41746-024-01404-1

**Published:** 2024-12-28

**Authors:** Brian C. Davis, Kai-Chun Lin, Sarah Shahub, Annapoorna Ramasubramanya, Andrew Fagan, Sriram Muthukumar, Shalini Prasad, Jasmohan S. Bajaj

**Affiliations:** 1 Richmond Veterans Affairs (VA) Medical Center and Virginia Commonwealth University, Richmond, VA USA; 2 University of Texas at Dallas, Richardson, TX USA; 3 EnLiSense LLC, Allen, TX USA

**Keywords:** Translational research, Liver cirrhosis

## Abstract

Patients with cirrhosis have high systemic inflammation (TNFα, CRP, and IL-6) that is associated with poor outcomes. These biomarkers need continuous non-invasive monitoring, which is difficult with blood. We studied the AWARE sweat-sensor to measure these in passively expressed sweat in healthy people (*N* = 12) and cirrhosis (*N* = 32, 10 outpatients/22 inpatients) for 3 days. Blood CRP, TNFα, IL6, levels, and liver function and quality of life were measured. We found that CRP, TNFα, and IL6 were correlated in sweat and serum among both groups and were evaluated in inpatients versus outpatients/controls. IL6 is associated with lower transplant-free survival. Sweat monitoring nocturnal CRP/IL6 elevations in cirrhosis versus controls. Outpatients with cirrhosis had inflammation levels that elevated during the evening and peaked towards the early night periods. The levels start to fall much later at night and early morning. These data suggest that further investigation of continuous measurement of sweat biomarkers in cirrhosis is warranted.

## Introduction

Liver cirrhosis is the end stage of chronic liver injury from conditions such as alcohol use disorder, viral hepatitis, and metabolic dysfunction, which is a major cause of morbidity and mortality worldwide^1–5^. Cirrhosis consists of a subclinical “compensated” stage, where liver function is largely preserved, then followed by a more advanced “decompensated” stage characterized by clinical manifestations such as infections, hepatic encephalopathy (HE), variceal bleeding, and ascites—resulting in hospitalizations and death^6–8^.

Systemic inflammation represents the major pathophysiological phenomenon in the progression of cirrhosis from the compensated to decompensated state^9–14^. Translocation of bacterial components known as pathogen-associated molecular patterns (PAMPs) from the intestinal lumen into the portal circulation leads to chronic overactivation of the innate immune system by stimulating the production of pro-inflammatory cytokines such as interleukin-6 (IL6), tumor necrosis factor alpha (TNFα), interferons, and others^12^. These cytokines play a key role in the host response to infections, but overstimulation may lead to further organ damage and secondary infections via an anti-inflammatory compensatory response and immune exhaustion^15,16^. C-reactive protein (CRP), an acute phase reactant, is elevated in the serum of patients with cirrhosis^17^. Increased serum levels of tumor necrosis factor alpha (TNFα) and interleukin-6 (IL6) are also found in cirrhosis^18–20^. Therefore, numerous studies have linked inflammatory markers to poor outcomes in decompensated cirrhosis and the development of multi-organ failure, termed acute on chronic liver failure (ACLF)^21–23^.

Emerging evidence links systemic inflammation and sleep disturbance in the general population and sleep issues are quite common in people with cirrhosis^24–26^. Systemic inflammation worsens neuropsychiatric test results and severity of HE episodes^9,10^. Furthermore, significant sleep disturbances have been described in covert or minimal HE patients^27^. Therefore, changes in sleep architecture may be of clinical significance and portend worsening of underlying systemic inflammation^28^.

Despite the research and advances in the systemic inflammation theory of cirrhosis progression, current clinical practice does not involve measuring cytokines and clinical guidelines do not support obtaining biomarkers to make clinical decisions^29^. First, these serum tests are invasive. Second, it is unclear of the optimal time(s) and impact of cirrhosis inflammatory biomarkers and their differential rhythmicity. Third, interpretation can be a challenge, and cut-offs may vary depending on the reference lab. Thus, the current standard of care involves obtaining cultures to rule out infection, which often takes several days to result and can lead to delays in diagnosis and treatment^6,15^.

**Fig. 1:**
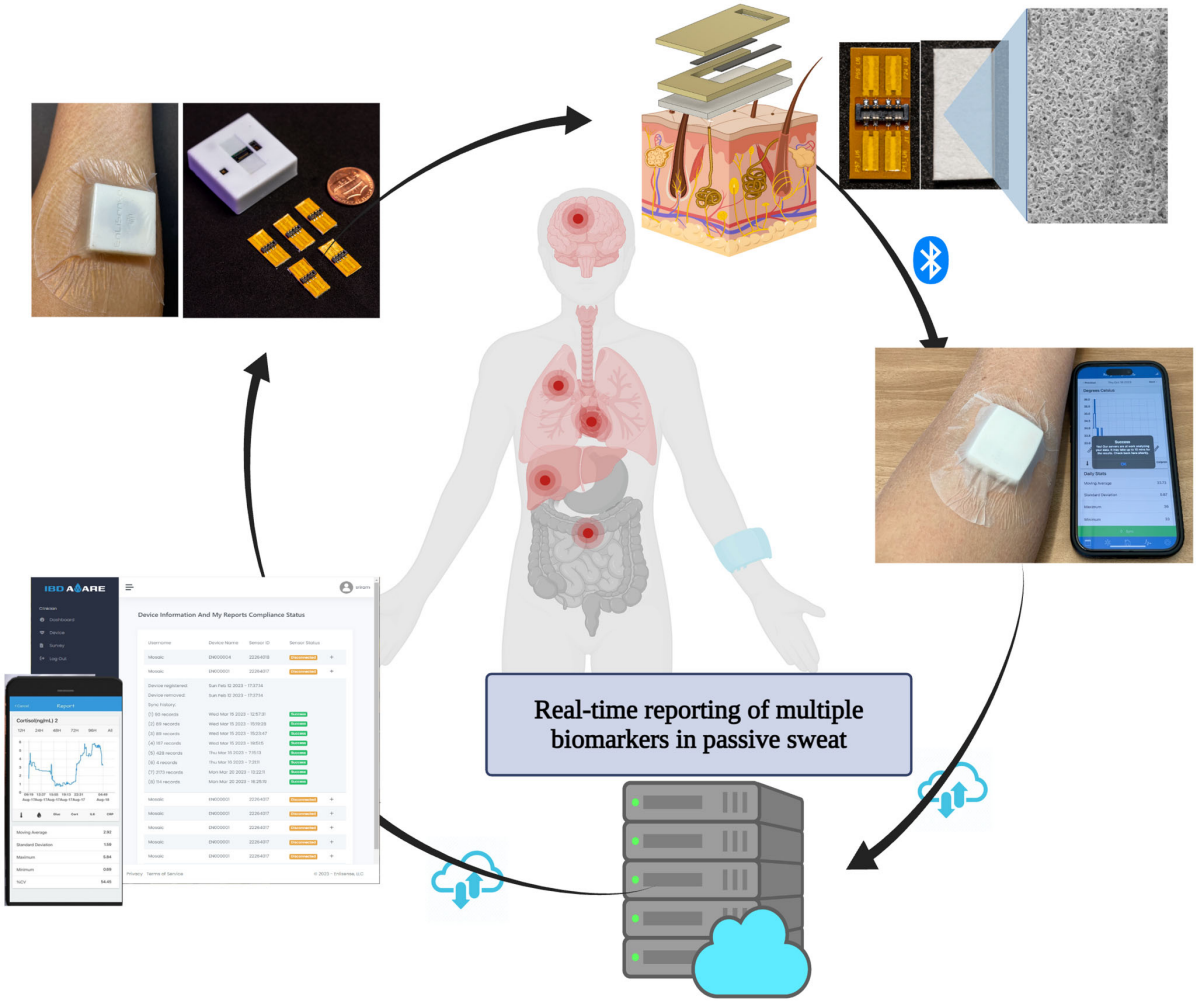
Sweat AWARE perspiration-based biosensing platform. This platform non-invasively detects inflammation levels and changes over time from sweat in a passive manner. The collected data is transmitted instantly through Bluetooth to the user’s smartphone and subsequently sent to the cloud server. This facilitates the immediate reporting of biomarker levels to both patients and healthcare providers. Figure created in BioRender. https://BioRender.com/y97p747.

The use of noninvasive sweat sensors to detect metabolites, ions, and pH has shown utility beyond intermittent blood draws in health and disease^30,31^. A similar detection of sweat cytokines at more frequent intervals could shed greater light into the pathophysiological basis of inflammation in cirrhosis for potential identification of high-risk patients, rather than intermittent blood sampling^32–34^. In addition, sweat-based technologies have been used for the management of chronic diseases such as cystic fibrosis as well as for closing the clinical loop through therapeutic drug monitoring^35,36^. A novel, Sweat AWARE perspiration-based biosensing platform (Fig. 1) that non-invasively detects inflammation levels and changes over time, would serve as an important interface between the patient and provider to improve the prediction of outcomes by linking these with patient reported-outcomes (PROs). The AWARE sweat sensor has been shown to track systemic inflammation markers in sweat among patients with other gastrointestinal disorders such as inflammatory bowel disease, but its application in cirrhosis is unclear^37^. The primary aim of this study was to characterize common cytokines in sweat such as TNFα, IL6, and CRP^38,39^. We also hypothesized that the AWARE sweat sensor would delineate diurnal patterns among subjects with cirrhosis due to the continuous measurement of cytokines when compared with healthy controls, these sweat cytokines signatures may vary based on inpatient or outpatient clinical status and would be linked with cognitive testing and outcomes in these patients.

## Results

A total of 12 controls (no-cirrhosis) and 32 veterans with cirrhosis (22 inpatients [IP] and 10 outpatients [OP]) were included in the study. Analyses were performed between groups at baseline as well as within groups over time. The median age of OP with cirrhosis was 64 years (range 42–74) and the median age of IP with cirrhosis was 65 years (range 35–77). Most cirrhosis subjects were male (100% outpatient and 91% outpatient) and half of each cirrhosis cohort identified as non-white. (Table 1)

**Table 1:** Baseline Characteristics in Study Population of healthy controls (no-cirrhosis) and cirrhosis subjects sweat biomarkers grouped by time of day (Morning vs. Evening vs. Night)

Parameter	Control, No-cirrhosis (*n* = 12)	Outpatient cirrhosis (*n* = 10)	Inpatient cirrhosis (*n* = 22)	*P*-value between cirrhosis groups
Age (years; median; range)	44.5 (40–47)	64; 42–74	65; 35–77	0.02
Sex (% male)	67%	100%	91%	0.91
Race (% white)	0%	50%	50%	0.82
Decompensated (%)	-	90%	82%	0.28
**Enrollment serum and sweat biomarkers**				
Serum CRP (mean, mg/dL)	-	0.48 ± 0.37	2.60 ± 2.20	<0.0001
Sweat CRP. Morning (mean, pg/mL)	1835.50 ± 1492.90	1685.33 ± 1062.72	2672.72 ± 1363.90	<0.0001
Sweat CRP. Evening (mean, pg/mL)	1423.99 ± 1113.26	2011.91 ± 1396.74	1300.98 ± 479.88	<0.0001
Sweat CRP. Night (mean, pg/mL)	1858.20 ± 1673.31	4063.02 ± 1458.81	3010.48 ± 1522.54	<0.0001
Serum IL6 (mean, pg/mL)	-	2.13 ± 1.53	24.5 ± 18.2	<0.0001
Sweat IL6, Morning (mean, pg/mL)	3.87 ± 2.37	4.99 ± 3.12	7.05 ± 4.40	<0.0001
Sweat IL6, Evening (mean, pg/mL)	3.33 ± 1.98	4.72 ± 3.09	5.08 ± 2.53	0.0002
Sweat IL6, Night (mean, pg/mL)	3.77 ± 2.07	10.16 ± 5.53	7.17 ± 3.71	<0.0001
Serum TNFα (mean, pg/mL)	-	4.22 ± 1.46	11.02 ± 8.04	0.004
Sweat TNFα, Morning (mean, pg/mL)	4.92 ± 1.76	6.01 ± 1.37	6.38 ± 1.68	<0.0001
Sweat TNFα, Evening (mean, pg/mL)	4.82 ± 1.64	6.00 ± 1.35	6.27 ± 1.47	<0.0001
Sweat TNFα, Night (mean, pg/mL)	4.71 ± 1.76	6.64 ± 1.59	6.38 ± 1.68	<0.0001

Blood samples were not collected in healthy controls. Data are presented as mean ± standard deviation unless otherwise noted.

*CRP* c-reactive protein, *I6* interleukin-6, *TNFα* tumor necrosis factor alpha.

All inpatients were admitted for cirrhosis-related complications with a mean length of stay 5.5 ± 0.81 days. Sixteen of the subjects had alcohol-related liver disease, nine with viral hepatitis, and seven with metabolic dysfunction as the primary etiology of cirrhosis. Fourteen IPs were given antibiotics and seven had documented infections: three urinary tract infections, one bacteremia, one Helicobacter pylori, one *S. aureus* hand wound infection, and one Coronavirus disease-2019 infection. Four inpatients had overt HE on admission. SIP was administered to 9 of 10 outpatients and 15 of 22 inpatients—SIP total, physical, and psychosocial scores were all higher in the inpatient cohort. Liver function, as measured by MELD-Na, was significantly worse on each day of the study in the inpatient group. The transplant-free survival rate after one year was 0.56 and significantly lower in the inpatient group (Table 2).

**Table 2:** Inpatients with cirrhosis have worse liver function and functional status than outpatients

	Outpatient Cirrhosis (*n* = 10)	Inpatient Cirrhosis (*n* = 22)	*P*-value
Total SIP	14.8 ± 8.3 (*n* = 9)	32.2 ± 16.6 (*n* = 15)	0.005
Physical SIP	12.6 ± 7.4	34.5 ± 20.2	0.002
Psychosocial SIP	13.4 ± 9.1	23.3 ± 21.8	0.16
Day 0 MELD-Na	9.4 ± 2.2	22.1 ± 10.6	<0.0001
Day 1 MELD-Na	9.7 ± 2.5	23.4 ± 10.4	<0.0001
Day 2 MELD-Na	9.1 ± 1.9	26.6 ± 9.7	<0.0001
Transplant-free survival (1 year)	9 (90%)	9 (41%)	0.001

Data are presented as mean ± SD unless otherwise noted. The SIP scores are a quality-of-life summary assessments and higher scores indicate lower quality of life. The MELD-Na score ranges from 6-40 with higher scores associated with higher risks of short-term mortality.

*HE* hepatic encephalopathy, *SIP* sickness impact profile, *MELD-Na* Model for End Stage Liver Disease Sodium.

**Fig. 2:**
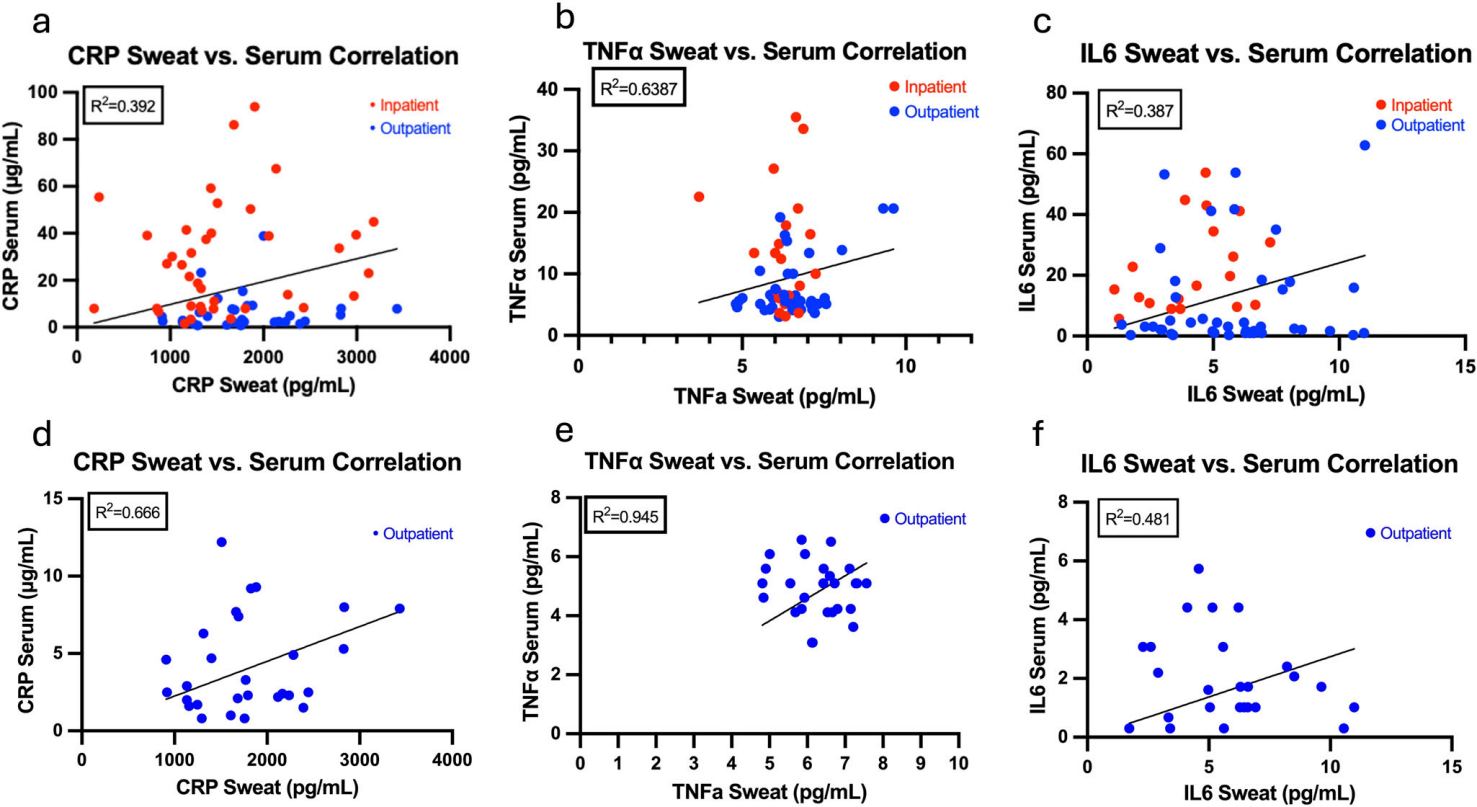
Coefficient of determination (*R*^2^) between CRP, TNFα, and IL6 in serum and sweat. These were measured in serum (lab-based) and sweat (2 h average of sweat CRP levels and 30 min average of sweat TNFα and sweat IL6 levels from the Sweat AWARE device) were highly significant across groups. **a**–**c** display data for both inpatients and outpatients, while **d**–**f** show data for outpatients only. Serum CRP levels in the outpatient group were below 15 µg/mL, whereas levels in the inpatient group reached up to 100 µg/mL.

The coefficient of determination (*R*^2^) of sweat and serum biomarkers was examined across different cirrhosis groups (Fig. 2a–f). Subjects in the inpatient group exhibited higher levels of each biomarker, regardless of the fluid source. The overall *R*² for CRP was 0.392 (Fig. 2a), while the *R*² for CRP for the outpatient group improved to 0.666 (Fig. 2d). Subjects in the outpatient group had serum CRP expression levels below 15 μg/mL, while subjects in the IP group had serum CRP expression levels up to 100 μg/mL. Higher serum CRP values did not correspond to a significant increase in sweat CRP levels. A similar pattern of differences in the coefficient of determination between serum and sweat levels was observed with TNF-α (Fig. 2b, e) and IL6 (Fig. 2c, f) biomarkers. These differences in the coefficient of determination between serum and sweat levels could be attributed to the optimization of the dynamic range of the sweat sensor assay performance for each of the biomarkers respectively. The Sweat AWARE device is designed to enable patient patient-centered clinical decision support system and is optimized for use as a remote patient monitoring system. The current dynamic range of the sweat sensor assay performance matching the inflammation levels of outpatient subjects would make the device optimal for primary use in a remote outpatient monitoring setup.

### Clinical outcomes and sweat analysis

**Fig. 3:**
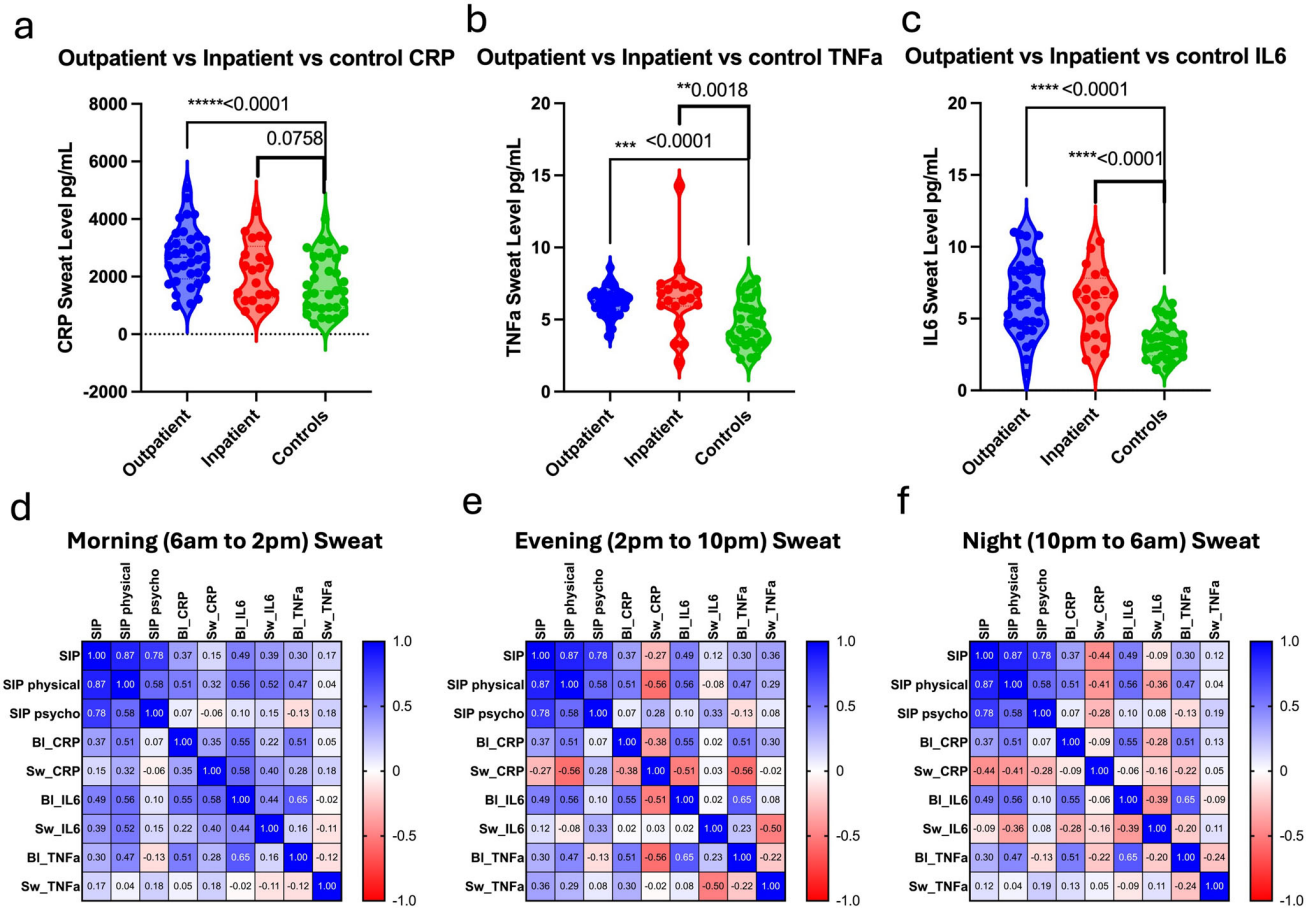
Sweat values and relationship with inpatient status and Sickness Impact Profile (SIP). In **a**–**c**, sweat biomarker averages (CRP, IL6, and TNFα) were compared across clinical groups and controls. All sweat levels of CRP, IL6, and TNFα were lower in the control group compared to both outpatient and inpatient groups. In **d**–**f**: correlation heatmap analysis of sweat and serum biomarker levels across different groups. Sickness Impact Profile (SIP) scores showed both positive and negative correlations with inflammatory markers (CRP, IL6, TNFα) in sweat based on the time of day. The heatmap analysis indicated a positive association between morning sweat CRP and IL6 levels and SIP physical quality-of-life scores.

When sweat biomarker averages were compared among clinical status and controls, all sweat CRP, IL6, and TNFα control levels were lower compared to outpatient and inpatient groups. (Fig. 3a–c). The measurements of CRP, IL6, and TNFα via the Sweat AWARE device were assessed to determine its utility in classifying inpatient and outpatient individuals versus control. The Sweat AWARE device effectively distinguished the healthy control group from outpatient cirrhosis based on the measured sweat biomarkers. The analysis of sweat and serum biomarker levels in patients who received antibiotics or had an infection, and those who did not, are shown in Supplementary Figs. 1–4. Due to a limited sample size, no statistically significant differences were observed in either sweat or serum biomarker levels (CRP, TNF-α, or IL6) between patients who received antibiotics and those who did not.

Correlation heatmap analysis of sweat and serum biomarker levels was examined in these groups (Fig. 3d–f). Sweat levels were averaged by time of the day, in three parts as Morning sweat levels between 6 am and 2 pm; Evening sweat levels between 2 pm and 10 pm; and Night sweat levels between 10 pm and 6 am. Sickness Impact Profile (SIP) scores showed both positive and negative correlations with inflammatory markers (CRP, IL6, TNFα) in sweat based on the time of the day. Moderately positive correlations were found for inflammatory marker levels in serum to sweat inflammatory levels for CRP and IL6 during morning time periods between 6 am and 2 pm, while a slight negative correlation was found for the serum to sweat TNFα levels. In contrast, moderately negative correlations were found between serum and sweat inflammatory levels for all 3 biomarkers for the Evening time periods between 2 pm and 10 pm and for the Nighttime periods between 10 pm and 6 am. It should be noted that blood sampling in the study and SIP scores were all done during the morning time period.

Correlation heatmap analysis suggested a positive association with morning time period sweat CRP and IL6 and SIP physical quality of life. Serum CRP had moderate correlations with SIP physical (0.51), serum IL6 (0.56), and serum TNFα (0.47). Morning time period sweat CRP had moderate correlations with SIP physical (0.32), serum IL6 (0.58), serum CRP (0.35), and serum TNFα (0.28). Serum IL6 had moderate to strong correlations with SIP physical (0.56), serum CRP (0.55), and serum TNFα (0.65). Morning time period sweat IL6 had moderate correlations with SIP physical (0.52), serum CRP (0.22), and serum IL6 (0.44). Serum TNFα had moderate correlations with SIP physical (0.47), serum CRP (0.51), and serum IL6 (0.65). Morning time period sweat TNFα showed very low correlations with all the other variables.

Overall, Inflammatory markers (CRP, IL6, TNFα) in serum generally show moderate correlations with the SIP physical subscale, suggesting a potential link between inflammation and physical symptoms. Inflammatory markers CRP and IL6 in morning sweat had generally moderate correlations with other variables, indicating that changes in these markers may reflect the SIP measures.

**Fig. 4:**
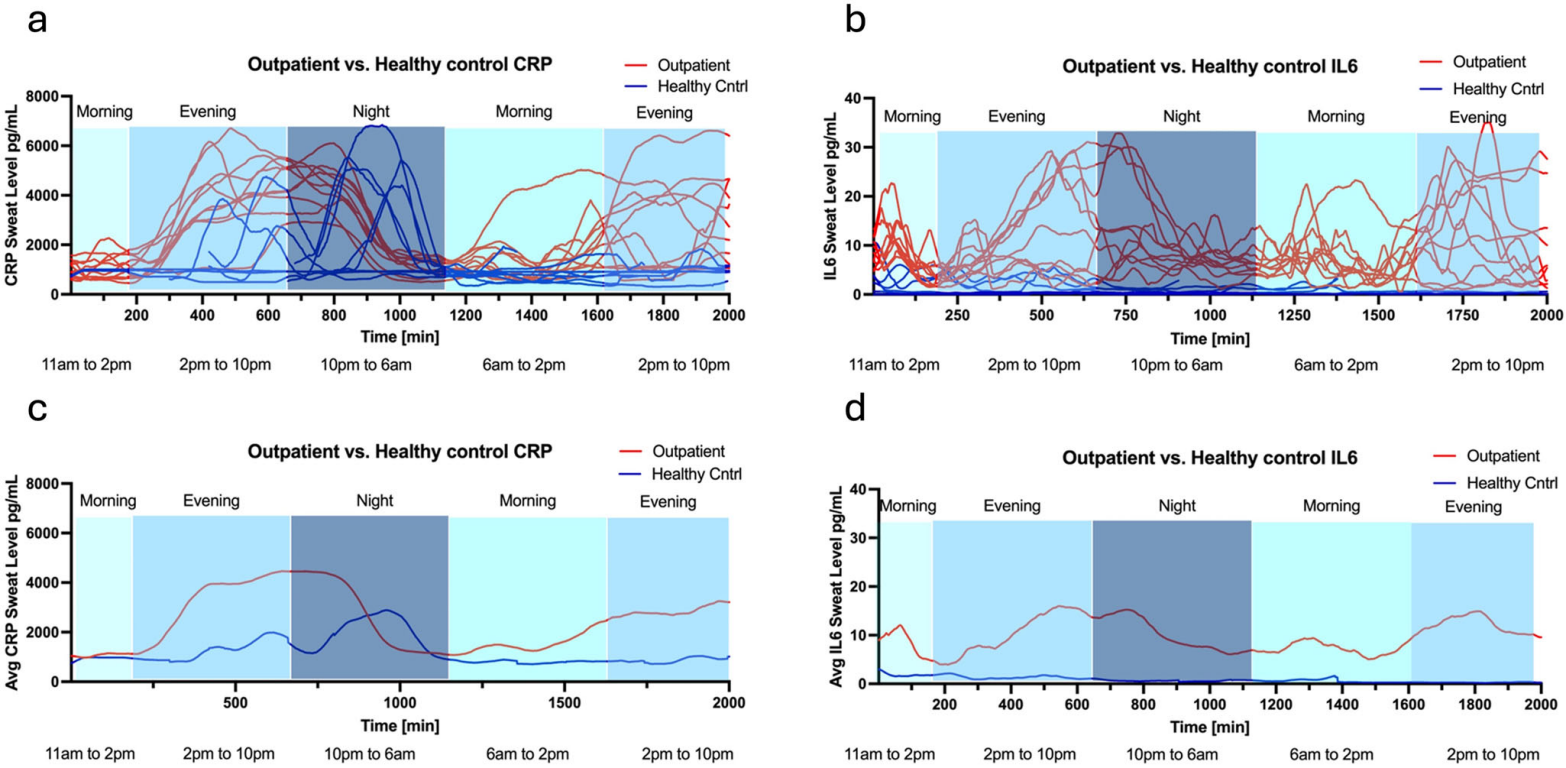
Temporal plots of sweat inflammatory biomarkers. **a**, **b** The temporal plots of CRP and IL6 for all subjects are displayed. **c**, **d** show the average values for all subjects, highlighting trends over different time periods. Outpatients with cirrhosis consistently exhibited elevated sweat inflammation levels starting in the evening and peaking in the early night periods.

Outpatients with cirrhosis when analyzed independently were consistently found to have sweat inflammation levels starting to elevate during the evening periods and peaking towards the early night periods. The sweat inflammation levels start to fall much later in the night periods and early morning periods. Moderately positive correlations were found for inflammatory marker levels in sweat to SIP scores for the evening time periods between 2 pm and 10 pm and moderately negative correlations for the morning time periods between 6 am and 2 pm. The correlations were mixed in the night periods between 10 pm and 6 am. These trends can be clearly seen in the temporal plots of the sweat inflammatory biomarkers (Fig. 4a–d).

**Fig. 5:**
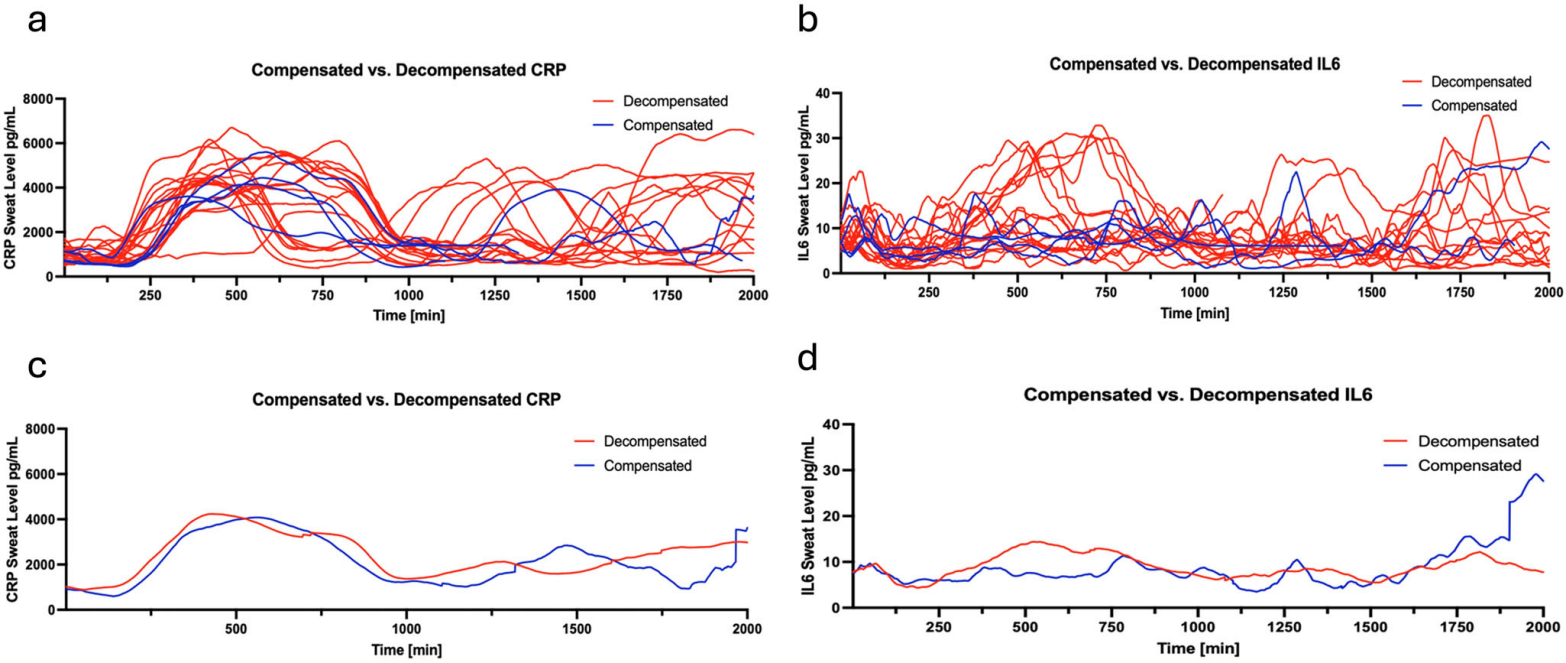
Sweat biomarkers temporal plots in cirrhosis cohort based on clinical status. Compensated (*n* = 5) was defined as clinically stable without HE, EVB, ascites, jaundice, or HRS. Decompensated status (*n* = 27) was defined as history or evidence of liver clinical events at study enrollment. **a**, **b** Sweat CRP and IL6 levels are plotted at 1 min intervals in subjects with decompensated (red) and compensated cohort (blue). Each line represents a subject’s sweat values over the follow-up period. **c**, **d** The mean sweat CRP and IL6 levels for the cohort of decompensated (red) and compensated (blue) over the follow-up period. CRP c-reactive protein, IL6 interleukin-6, TNF tumor necrosis factor alpha.

**Fig. 6:**
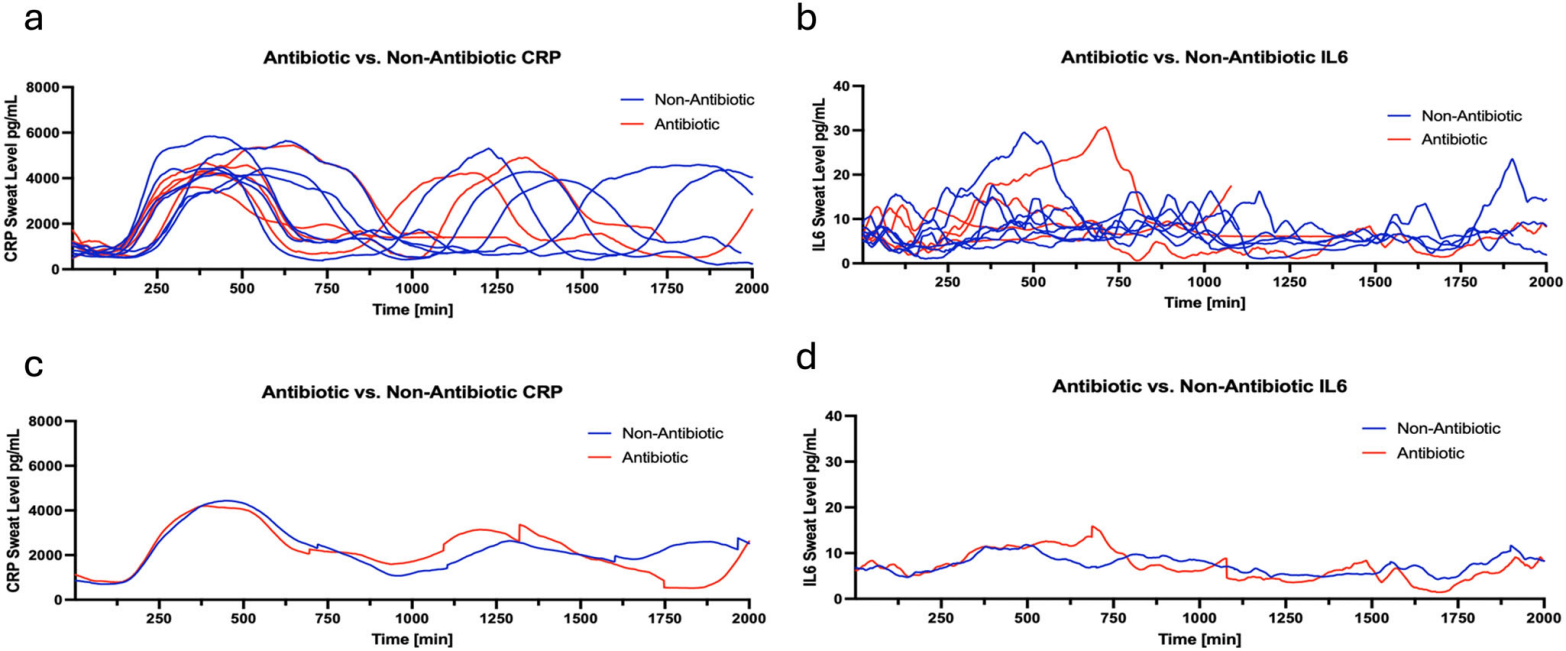
Sweat biomarker temporal plots in inpatients with cirrhosis based on antibiotic exposure. Fourteen subjects received antibiotics and eight did not. **a**, **b** Sweat CRP and IL6 levels are plotted at 1 min intervals in subjects receiving antibiotics (red) and non-antibiotic cohort (blue). Each line represents a subject’s sweat values over the follow-up period. **c**, **d** The mean sweat CRP and IL6 levels for the cohort of subjects with antibiotic (red) and non-antibiotic cohort (blue) over the follow-up period.

**Fig. 7:**
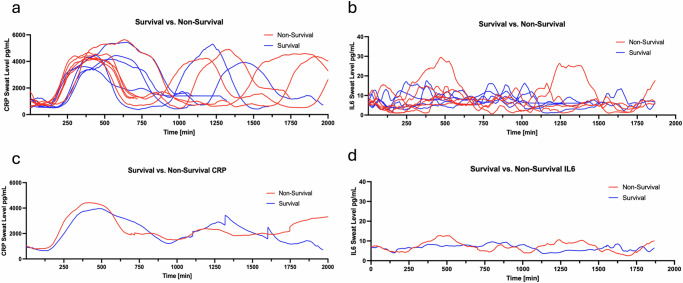
Sweat biomarker temporal plots in subjects based on transplant-free survival. **a**, **b** Sweat CRP and IL6 levels are plotted at 1 min intervals in subjects with non-survival (red) and survival cohort (blue). Each line represents a subject’s sweat values over the follow-up period. **c**, **d** The mean sweat CRP and IL6 levels for the cohort of non-survival (red) and survival (blue) over the follow-up period.

Among compensated versus decompensated cirrhosis, the average decompensated sweat biomarker values measured during the study period were slightly higher in both CRP and IL6 (Fig. 5a–d). In subjects who received antibiotics, the sweat CRP and IL6 levels were lower than subjects who did not receive antibiotics, where the area of curve of antibiotic of CRP is 4,384,945 and non-antibiotic is 4,557,502 (Fig. 6a–d). In subjects who died or received a liver transplant, the admission sweat CRP and IL6 levels were elevated compared with subjects who survived (Fig. 7a–d).

### Differential rhythmicity characteristics of sweat biomarker expression

**Fig. 8:**
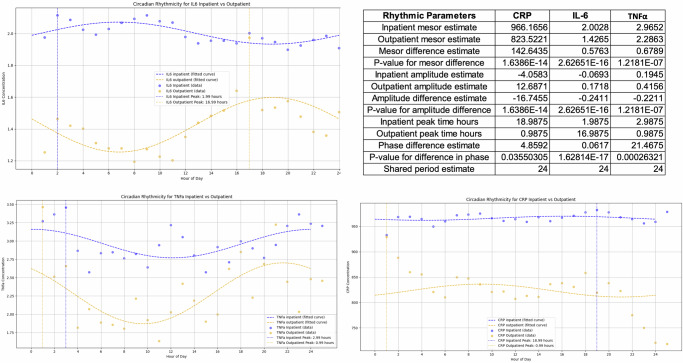
The CircaCompare analysis. This is an estimation of rhythmic parameters [mesor (the rhythm-adjusted mean level of a response variable around which a wave function oscillates), amplitude, and phase], and simultaneously testing for statistical significance in all three parameters between two or more groups of datasets, i.e. Outpatients cohort and Inpatients cohort.

**Fig. 9:**
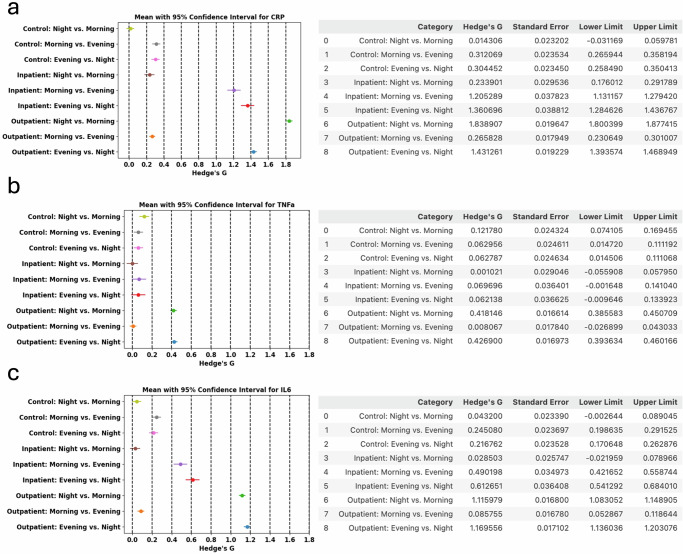
Hedge’s G analysis of the effect size of the sweat biomarker expression over time. This is segmented by the time of the day i.e., Morning, Evening, and Night and by cohort i.e. Control, Inpatients, and Outpatients for CRP (**a**), TNFα (**b**), and IL6 (**c**). A relatively small or no-differential rhythm effect is observed in the Control cohort across all sweat biomarkers, while a significant effect is observed for outpatients for CRP and IL6 expression in sweat. Inpatients also show a differential rhythm expression in sweat CRP and IL6 albeit to a much smaller effect than outpatients. Sweat TNFα expression across all patient cohorts was smaller than that observed with sweat CRP and IL6 expressions respectively.

Any physiological or behavioral characteristic exhibit differential rhythm and quantifying this rhythm has inherent value^40^. Inflammation is elevated due to chronic conditions and the relative changes in the inflammation levels over time are due to the illness states and the physiological state of the subject. Diurnal refers to a characteristic elevation of biomarker expression mainly during the daytime while nocturnal refers to a characteristic elevation of biomarker expression mainly during night time^41^. CircaCompare (Fig. 8) and Hedge’s G (Fig. 9) were used to determine the differential rhythmic expression of the sweat inflammatory biomarker levels over time. CircaCompare provides a means to quantify and statistically support differences between rhythms, specific to the characteristic desired (mesor, amplitude, and phase) between two datasets collected over a 24-h period. We ran the CircaCompare analysis between the sweat biomarker measurements. The results establish that the differential rhythmicity in the outpatient inflammation levels is higher in amplitude than that in inpatients across all three sweat biomarkers even though outpatients express lower sweat inflammation levels than inpatients. CRP and TNFa were also different between decompensated and compensated patients (Supplementary Fig. 5) but none of these were different when inpatients with and without infections, or those who survived or not were compared (Supplementary Figs. 6 and 7).

The Hedge’s G statistic expresses the difference of the means in units of the pooled standard deviation. It is typically used in the context where one of the samples is a control sample. That is, we are interested in the effect size of the sample being analyzed relative to a control sample. A Hedge’s G value of 0.2 or lower (small effect); 0.2–0.5 (medium effect); and 0.8 or greater (large effect). In this case, we use this Hedge’s G statistic to compare the means of the sweat inflammatory biomarker levels averaged by the time of the day, in three parts as Morning sweat levels between 6 am and 2 pm; Evening sweat levels between 2 pm and 10 pm; and Night sweat levels between 10 pm and 6 am and comparing between subjects with cirrhosis i.e., inpatients and outpatients who are inflamed due to cirrhosis and healthy control subjects with no inflammation.

The Hedge’s G for sweat CRP and IL6 in Fig. 9a, c shows that the patients with cirrhosis have higher Hedge’s G value at night when compared to morning or evening periods hence, the significance of the biomarker is higher in the night. For TNFα the Hedge’s G values are all below 0.5 indicating that the effect size is low to medium as compared to CRP and IL6 which had Hedge’s G values above 0.8 indicating a larger effect size in CRP and IL6 expressions in sweat. From the Hedge’s G values for the three sweat biomarkers, it can be concluded that CRP and IL6 show a significant effect size between evening and night periods in cirrhosis subjects compared to morning periods, while the same significance in effect size is not observed in control subjects.

## Discussion

In this study, we demonstrated a novel sweat sensor device can safely be applied to outpatients and inpatients with cirrhosis and generate discriminatory data when compared to healthy controls. We showed that serum and sweat inflammatory markers are correlated in patients with cirrhosis. We found that decompensated patients had dampened diurnal variation of these cytokine levels compared to compensated patients and healthy controls. Sweat-based inflammatory biomarkers on average were higher in infected patients and in those who had a poor transplant-free survival.

A key attribute of this study is the demonstration that continuous sweat monitoring illustrates important clinical manifestations of a chronic inflammatory disease state, such as cirrhosis. For instance, sleep cycle disturbances were first described in decompensated cirrhosis in the 1950s, and complaints such as difficulty falling asleep, insomnia, and excessive daytime sleepiness are quite common in the cirrhosis population^24,26,28,42,43^. However, the pathophysiology is complex and incompletely understood with various internal and external factors playing a role. For instance, the release of melatonin is disrupted in cirrhosis, which leads to higher rates of insomnia and excessive daytime sleepiness and naps^44,45^. Furthermore, co-morbid conditions such as sleep apnea also play a role in cirrhosis patients’ sleep disruption^46^. Emerging data show circadian disorders are linked with chronic inflammation^25^. Our study provides intriguing evidence that continuous noninvasive monitoring of inflammatory biomarkers may predict outcomes – as patients with cirrhosis had higher evening and nocturnal levels of sweat CRP and IL6 when compared with healthy controls.

Serum IL6 is associated with poor outcomes in patients with cirrhosis using blood levels in prior studies^11^. However, those studies have largely focused on blood levels at discrete timepoints without focusing on variations in levels over time. Cirrhosis is a disease of chronic inflammation requiring frequent hospitalizations and numerous studies have looked at serum biomarkers as predictors of inflammation and decompensation^47^. We chose sweat CRP, IL6, and TNFα as the target cytokines in this study based on previous research of serum studies linking these biomarkers to important clinical outcomes. Perdigoto et al.^48^ showed that serum CRP can predict infections in a prospective cohort of inpatients with cirrhosis. A recent meta-analysis documented serum IL6 may discriminant bacterial infections and predict hepatic encephalopathy in subjects with cirrhosis^49,50^ TNFα also elevated in decompensated cirrhosis and correlates with the severity of ACLF, although a clinical trial of anti-TNFα therapy did not show a benefit in patients with ACLF due to increased secondary infections^14,51^. However, the unique patterns of cytokines throughout the day in decompensated and more advanced patients compared to healthy people and outpatients could be more important in monitoring patients over time than one-time blood draws. This was found using complementary analysis of rhythmicity change (Circacompare) and relative change in cytokines compared to control values in specific cohorts (Hedge’s G). We did not show significant differences in infected or uninfected patients using both approaches likely due to the relatively small sample size and the time period that spanned days after adequate antibiotic therapy. This follows the differential rhythms in these cytokine levels and extends them into compensated and decompensated cirrhosis patients^52^.

In addition to survival and infections, investigating quality-of-life impairment and inflammation is critical in cirrhosis^47,53^. SIP inquiries about QOL over the last 24 h were administered in the morning, as was the blood draw. As expected more advanced patients (inpatients) had a worse QOL compared to outpatients. Interestingly, while there was a moderate correlation between morning blood inflammatory markers and SIP, this pattern was distinct across time periods in the sweat. Morning sweat inflammatory markers were more positively correlated with SIP but not the evening and nighttime levels. This differential rhythmic variation is important in case these questionnaires are administered during different times of the day.

Previous sweat sensor studies used healthy controls to identify potential chronic inflammatory markers and showed a correlation with serum and sweat CRP and IL6 as distinguishing factors in patients with inflammatory bowel disease (IBD)^37,54^. The Sweat AWARE device showed a direct correlation between serum and sweat markers among inpatients with IBD-related complications. Early data also suggest a pattern in sweat biomarkers among active IBD versus healthy controls.

This study had several limitations. The sample size was small as this was a pilot study, thus larger and more heterogeneous cohorts are needed to further define sweat inflammatory patterns. We were unable to analyze sweat data between compensated and decompensated outpatients based on the small sample size. Longer clinical outcome data are also needed, although we did show the SIP scores were worse among inpatients and correlated with sweat cytokines, and elevated IL6 was associated with reduced TFS. The sweat sensor data were collected for a short interval (up to 3 days) and longer monitoring may identify distinct patterns in the natural history of hospitalized patients. While the sweat sensor was noninvasive and easy to apply, further modification of the sensor to generate point-of-care results (i.e., wider dynamic range) would be useful to obtain clinical applicability as patients with cirrhosis express high levels of inflammation as observed by the serum levels analyzed in this study. Given the large amount of data produced by the AWARE sensor, future studies should apply deep-learning models for analysis. Finally, serum data were drawn daily as opposed to continuous monitoring with the sweat sensor so the correlations may be over or underestimated depending on the timing of the lab draw. We attempted to correct this discrepancy by trying to average sweat data over 2 h based on the half-life of each biomarker. Using these data, we could envisage the use of this technology in predicting inpatient and outpatient outcomes in patients with advanced cirrhosis and peri-transplant, specifically focusing on early detection of infection, rejection of the grafts, and progression to acute on chronic liver failure.

In conclusion, a novel, noninvasive device detects inflammatory cytokines in the sweat of patients with cirrhosis. The sweat biomarkers correlated with serum values and distinct sweat patterns are seen in outpatients and inpatients with cirrhosis. Sweat Inflammation biomarker levels are elevated in those with cirrhosis and follow a differential rhythmic behavior and there as differences in expression between outpatients and inpatients. Such an analysis would not have been possible to assess with serum collections. However, this is a limited sample size and these need to be validated in a larger cohort for a longer duration and by using machine learning analysis of these complex rhythms. Specifically, sweat cytokines are elevated in hospitalized patients who are at higher risk for infections. Elevated sweat IL6 may be an important predictor of mortality and larger studies are indicated to further characterize inflammatory sweat biomarkers in cirrhosis.

## Methods

We prospectively enrolled healthy controls, outpatients with cirrhosis, and inpatients with cirrhosis. Subjects were enrolled from the University of Texas at Dallas and Richmond VA Medical Center. All subjects needed to be >18 years of age and able to provide informed consent.

Healthy controls were recruited from the community and were free of chronic diseases and were not on prescription medications. For the two cirrhosis groups, cirrhosis was diagnosed by either biopsy, imaging, transient elastography, presence of varices and/or platelet count <150,000 and AST/ALT > 1 in chronic liver disease patients, or those with frank decompensating events [ascites, hepatic encephalopathy (HE), hepato-pulmonary syndrome, jaundice, variceal bleeding]. We excluded those with an unclear diagnosis of cirrhosis, with concomitant IBD, and those on immunosuppressive therapy. We only included subjects who could come in daily or be available daily for 3 days. Patients with cirrhosis without prior or current decompensating events (mentioned above) were considered compensated, while decompensated patients were those with current or prior decompensating events.

We recorded demographics, disease severity, course and complications, and concomitant medications at baseline and at each study visit. After consenting, we analyzed routine daily labs (basic metabolic and hepatic panel [BMP], complete blood count [CBC]), serum inflammatory markers (C-reactive protein [CRP], interleukin-6 [IL6], and tumor necrosis factor alpha [TNFα]) in those with cirrhosis. Patients with cirrhosis were also administered the Sickness Impact Profile (SIP), a generic quality-of-life (QOL) instrument that inquiries about daily function related to health over the last 24 h on the day of enrollment^55^. SIP consists of physical and psychosocial domains and a high SIP indicates poor QOL. Both SIP and blood draw were in the mornings. The sensor was then fitted to their arm, and subjects were added in a de-identified manner in the study iPad. Sensors were replaced every 24 h, and their capture of the data was checked before reloading.

During the study period, if the inpatients were considered stable for discharge, the study was continued as outpatients with daily return till day 3 post-enrollment. We did not withdraw those who developed infections or required antibiotics during the study period. Subjects who became confused during the study continued to have clinical data recorded but were withdrawn from active study participation. Subjects were followed for up to one year to calculate transplant-free survival i.e. percentage of subjects who did not die or undergo liver transplantation.

### Ethics declaration

Healthy control human subject studies were approved by the Institutional Review Board of the University of Texas at Dallas (IRB number UTD IRB 19–146) and Richmond VA Medical Center for patients with cirrhosis (IRB number 1649873 mIRB 02701 BAJAJ0028). All subjects signed informed consent.

### Sweat AWARE device

This comprises a replaceable sweat-sensing strip tailored for specific target biomarkers, affixed to a wearable electronic reader (Fig. 1). The authors have obtained written consent to publish the images. This reader translates the sensor’s impedance into a calibrated concentration of measured biomarker levels in sweat. The sensor response was measured through non-Faradaic electrochemical impedance spectroscopy (EIS), recording the resulting impedance at a frequency range of 100 Hz–1 KHz using a low sinusoidal input voltage of 1–100 mV. The sensor electrode underwent a functionalization process involving the application of a thiol cross-linker. This cross-linker was specifically chosen for its molecular properties. The opposite end of the cross-linker was meticulously bonded with a concentration of monoclonal capture antibodies, each tailored for the biomarkers CRP, IL6, and TNFα. This careful selection of monoclonal antibodies was deliberate, aiming to achieve a high degree of specificity in detecting the target biomarkers. We have previously outlined the fabrication process for both the Sweat AWARE device and sweat sensors. The sensor fabrication process has been adapted from Munje et al., and Jagannath et al., and has been described in detail previously^32,37^. When the Sweat AWARE device is applied to the patient’s arm, sweat is absorbed by the sensing strip. Biomarkers such as CRP, IL6, and TNFα present in the sweat selectively bind to their respective antibodies on the strip. Impedance measurements are then obtained and compared to the established dose-response curves, allowing for the quantification of CRP, IL6, and TNFα levels in the sweat. The Sweat AWARE device incorporates a calibration process that adjusts the sensor response based on different sweat rates and residual sweat accumulation. This calibration is optimized using a machine learning model, which integrates several key parameters, including the raw impedance signal, time, temperature, and relative humidity^56^. Data collected from human subject studies were used to refine this model. Measurements of sweat CRP, IL6, and TNFα were obtained on the body at one-minute intervals throughout each 24-h period. These recorded measurements were then averaged for every consecutive 2-h period, commencing from the start of each collection period. Following this, the averaged values were compared to serum levels to analyze the correlation between sweat and serum concentrations. For the correlation analysis, we used a 2-h average of sweat CRP levels and a 30-min average of sweat TNFα and IL6 levels to correlate with serum results. Temporal graphs of the actual and average levels of CRP, IL6, and TNFα were generated for all subjects, with categorization based on different subject groups.

### Statistical analysis

GraphPad Prism version 10.2.1 software was utilized to conduct analyses and generate figures. P-values were computed based on the average data of sweat and serum across all days. The Mann–Whitney test and one-tailed analysis were employed. Sweat value in this analysis was calculated by averaging sweat CRP, IL6, and TNFα measurements collected every 1-min. Additionally, a heatmap was generated using the correlation matrix function within the GraphPad analysis tool. Since this was a pilot experience, formal sample size analysis was not performed but prior experience of this sweat sensor in inflammatory bowel disease was used to guide enrollment^54,57^.

### Differential rhythmic patterns using CircaCompare

We performed two different methods for assessing the differences in rhythmic patterns in the sweat measurements across the three biomarkers. We performed the CircaCompare analysis, the chronobiology tool developed by Parsons et al. ^58^ for comparing patterns between groups of rhythmic data. CircaCompare provides a means to quantify and statistically support differences between rhythms, specific to the characteristic desired (mesor, amplitude, and phase) between two datasets collected over a 24-h period. The methods were taken from the source provided by the authors at https://github.com/RWParsons/circacompare/ and were applied to the datasets in this study. We ran the CircaCompare analysis between the sweat biomarker measurements of the outpatient and the inpatient cohorts, compensated/decompensated, those with/without infections, or those who survived or not.

### Differential rhythmic patterns using Hedge’s G

We also performed an assessment of the pattern differences using Hedge’s G statistics that are commonly used in effect size estimations. An effect size in statistics is a sample-based estimate of the number that quantifies the strength of the association between two variables in a population. It can be used to describe the value of a parameter for a fictitious population, the value of a statistic derived from a sample of data, or the equation that operationalizes the relationship between parameters and statistics and the effect size value. The correlation between two variables, the regression coefficient in a regression, the mean difference, or the likelihood that a certain event (like a heart attack) would occur are a few examples of effect sizes. In addition to serving as a supplement to statistical hypothesis testing, effect sizes are crucial for power analyses, sample size planning, and meta-analyses. The group of techniques for analyzing data related to effect sizes is known as estimate statistics. Hedge’s G is comparable to Glass’s G and Cohen’s D statistics. Usually, an experimental dataset and a control dataset are compared using these statistics. Hedge’s G is particularly useful for analyzing circadian rhythm differences.

Hedge’s G technique has (a) standardized effect size: It provides a standardized measure of the difference between two groups, accounting for both the difference in means and the variability in the data, (b) Sample size correction: The correction factor makes it especially suitable for smaller sample sizes, which is common in circadian studies, (c) Interpretable thresholds: The conventional thresholds (0.2 for small, 0.5 for medium, 0.8 for large effects) help interpret the biological significance of rhythm differences.

This analysis is particularly valuable for Comparing different chronotypes (e.g., morning vs. evening types), assessing intervention effects on circadian rhythms, studying circadian misalignment in different populations, and quantifying phase shifts or amplitude changes.

We adopted in here the Hedge’s G technique to assess the circadian characteristics of the sweat biomarker expression by calculating the impact magnitude of the mean difference across the 24-h time period i.e., Morning (6 am–2 pm), Evening (2 pm–10 pm), and Night (10 pm–6 am) and by subject cohorts i.e., Control, Inpatient, and Outpatient for each of the sweat biomarkers measured. The 24-h time period was divided into three 8-h periods by taking into consideration the relative half-life of the inflammatory biomarkers for assessing effect size. The Hedge’s G was calculated was calculated by comparing 2 time periods for each of the subject cohorts and using the formula as below^59^: *g* = (*y1*-*y2*)/sp, where, *y1* and *y2* are the standard means of the 2 samples and sp is the pooled standard deviation given by: sp= √(((*n1*-1) 〖*s1*〗^2 + (*n2*-1) 〖*s2*〗^2)/(*n1* + *n1*-2)).

## Supplementary information

Supplementary 12-3-24_KL JSB v2.1

## Data Availability

The datasets generated and/or analyzed during the current study are not publicly available due to IRB restrictions but are available from the corresponding author upon reasonable request.
