# Supplementary material for: A novel sweat sensor detects inflammatory differential rhythmicity patterns in inpatients and outpatients with cirrhosis
Source: NPJ Digit Med. 2024 Dec 28;7:382. doi: 10.1038/s41746-024-01404-1 (PMC11682110; doi:10.1038/s41746-024-01404-1)
Supplement: Supplementary file 1 — Supplementary 12-3-24_KL JSB v2.1 [file 41746_2024_1404_MOESM1_ESM.pdf]

Supplementary results:

Supplementary figures 1-4 show sweat and serum biomarker levels in patients who received antibiotics or had an infection, and those who did not, while figures 5-7 show Circacompare comparisons between compensated/decompensated, infection/not, and survived/not.

Supplementary Figure 1: Comparisons of sweat biomarkers in inpatients receiving antibiotics versus not.

None of the sweat biomarkers (CRP, TNFa, or IL6) demonstrate a statistically significant difference between subjects. Violin plots with blue (on antibiotics) and red (not on antibiotics) are shown with p-values showing non-significant results on a one-tailed analysis.

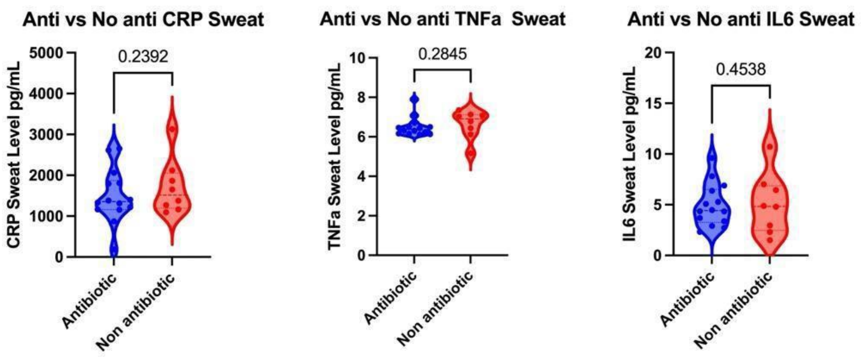

Supplementary Figure 2: Comparisons of serum biomarker levels in serum between inpatients who received antibiotics and those who did not.

None of the serum biomarkers (CRP, TNFa, or IL6) demonstrate a statistically significant difference between subjects. Violin plots with blue (on antibiotics) and red (not on antibiotics) are shown with p-values showing non-significant results on a one-tailed analysis.

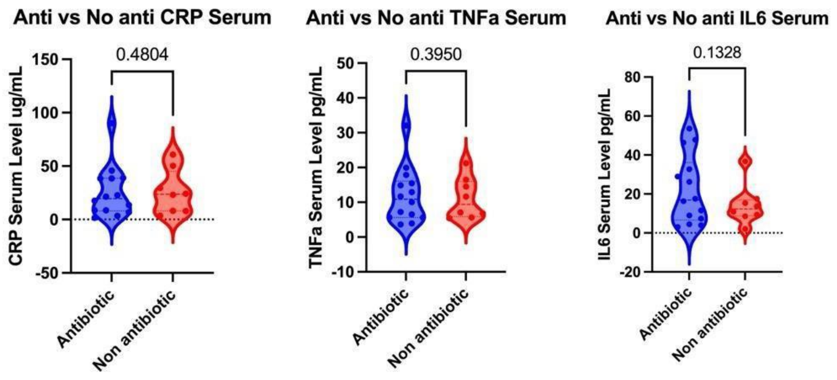

Supplementary Figure 3: Comparisons of sweat biomarkers in inpatients with infections versus not. None of the sweat biomarkers (CRP, TNFa, or IL6) demonstrate a statistically significant difference between subjects. Violin plots with blue (with infection) and red (not infected) are shown with p-values showing non-significant results on a one-tailed analysis.

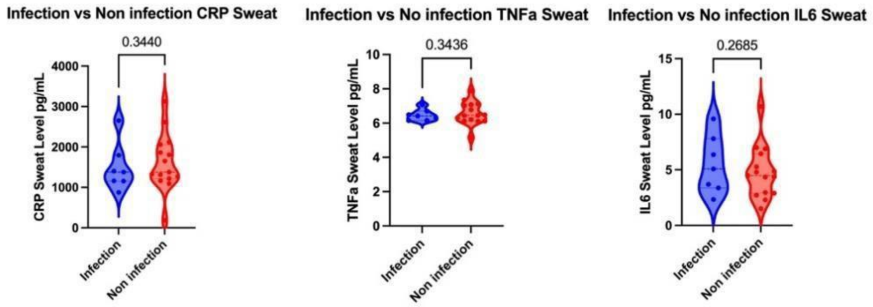

Supplementary Figure 4: Comparisons of serum biomarkers in inpatients with infections versus not. None of the serum biomarkers (CRP, TNFa, or IL6) demonstrate a statistically significant difference between subjects. Violin plots with blue (with infection) and red (not infected) are shown with p-values showing non-significant results on a one-tailed analysis.

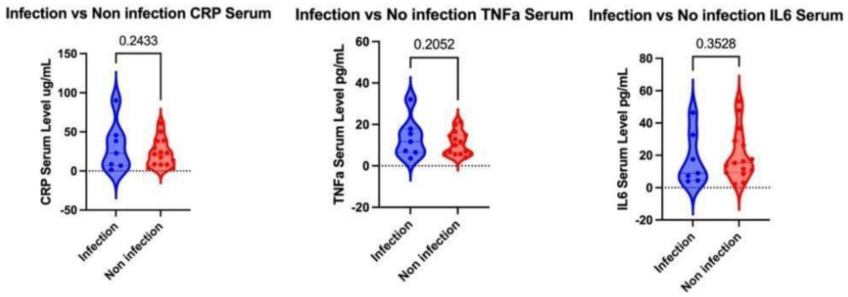

Supplementary Figure 5: Comparisons of analysis in Circacompare of sweat biomarkers in inpatients with compensated/decompensated. This is an estimation of rhythmic parameters [mesor (the rhythm-adjusted mean level of a response variable around which a wave function oscillates), amplitude and phase], and simultaneously testing for statistical significance in all three parameters between two groups of datasets, i.e. compensated/decompensated cohort. CRP and TNFa show a significant amplitude difference. Top panel is CRP, middle panel is IL-6 and bottom panel is TNFa.

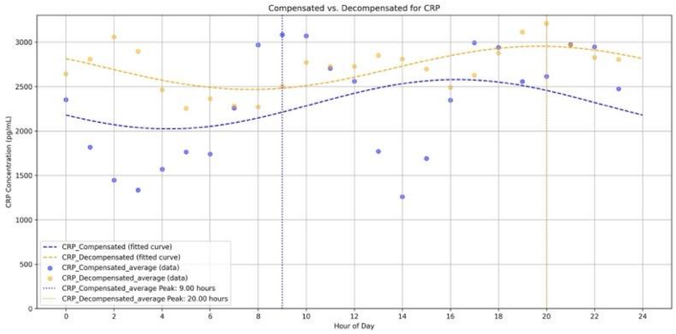

Metric	Value
Compensated amplitude estimate	-277.773
Decompensated amplitude estimate	243.8033
Amplitude difference estimate	-521.576
P-value for amplitude difference	0.00525
Compensated peak time hours	9
Decompensated peak time hours	20
Phase difference estimate	15.43604
P-value for difference in phase	0.548316

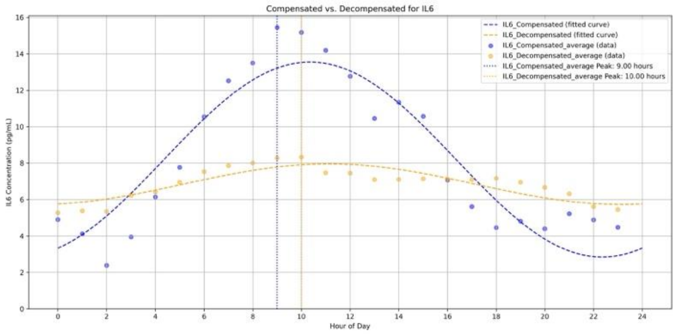

Metric	Value
Compensated amplitude estimate	-5.35321
Decompensated amplitude estimate	-1.11079
Amplitude difference estimate	-4.24242
P-value for amplitude difference	0.131399
Compensated peak time hours	9
Decompensated peak time hours	10
Phase difference estimate	0.789639
P-value for difference in phase	0.038122

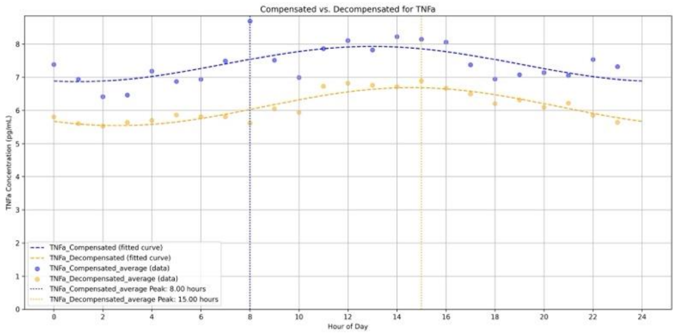

Metric	Value
Compensated amplitude estimate	-0.53094
Decompensated amplitude estimate	-0.57132
Amplitude difference estimate	0.040382
P-value for amplitude difference	5.35E-11
Compensated peak time hours	8
Decompensated peak time hours	15
Phase difference estimate	1.61526
P-value for difference in phase	0.355458

Supplementary Figure 6: Comparisons of analysis in Circacompare of sweat biomarkers in inpatients with antibiotic or not. This is an estimation of rhythmic parameters [mesor (the rhythm-adjusted mean level of a response variable around which a wave function oscillates), amplitude and phase], and simultaneously testing for statistical significance in all three parameters between two groups of datasets, i.e. antibiotic/non-antibiotic cohort. No significant differences in amplitude or phase between the 2 groups across all 3 biomarkers. Top panel is CRP, middle panel is IL-6 and bottom panel is TNFa.

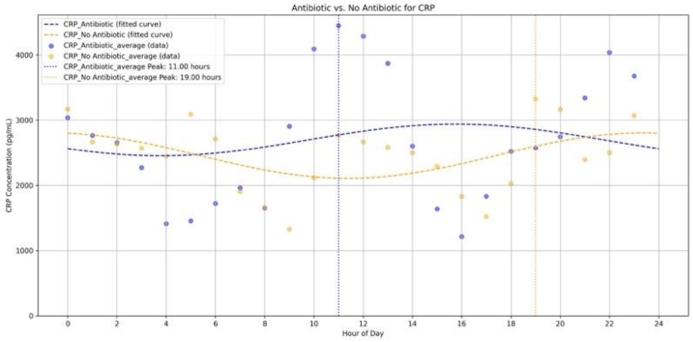

Metric	Value
Antibiotic amplitude estimate	-242.278
No Antibiotic amplitude estimate	347.18
Amplitude difference estimate	-589.458
P-value for amplitude difference	0.29968
Antibiotic peak time hours	11
No Antibiotic peak time hours	19
Phase difference estimate	19.60556
P-value for difference in phase	0.70224

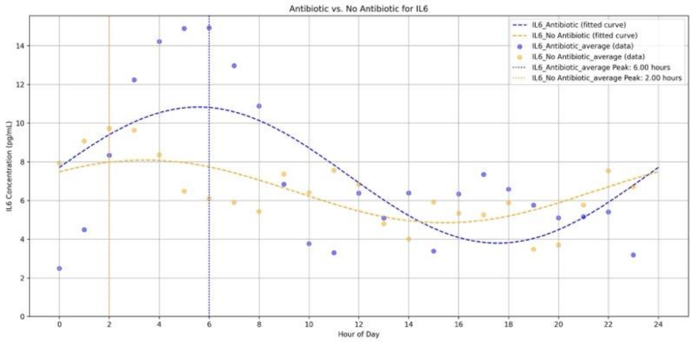

Metric	Value
Antibiotic amplitude estimate	3.515815
No Antibiotic amplitude estimate	1.623936
Amplitude difference estimate	1.891878
P-value for amplitude difference	0.337928
Antibiotic peak time hours	6
No Antibiotic peak time hours	2
Phase difference estimate	21.85782
P-value for difference in phase	0.220993

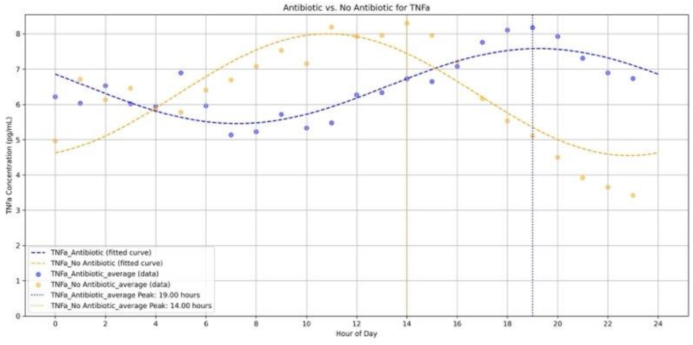

Metric	Value
Antibiotic amplitude estimate	1.064329
No Antibiotic amplitude estimate	-1.72632
Amplitude difference estimate	2.790653
P-value for amplitude difference	0.483173
Antibiotic peak time hours	19
No Antibiotic peak time hours	14
Phase difference estimate	3.607521
P-value for difference in phase	0.792109

Supplementary Figure 7: Comparisons of analysis in Circacompare of serum biomarkers in inpatients with survival/non-survival. This is an estimation of rhythmic parameters [mesor (the rhythm-adjusted mean level of a response variable around which a wave function oscillates), amplitude and phase], and simultaneously testing for statistical significance in all three parameters between two groups of datasets, i.e. survival/non-survival cohort. No significant differences in amplitude or phase between the 2 groups across all 3 biomarkers. Top panel is CRP, middle panel is IL-6 and bottom panel is TNFa.

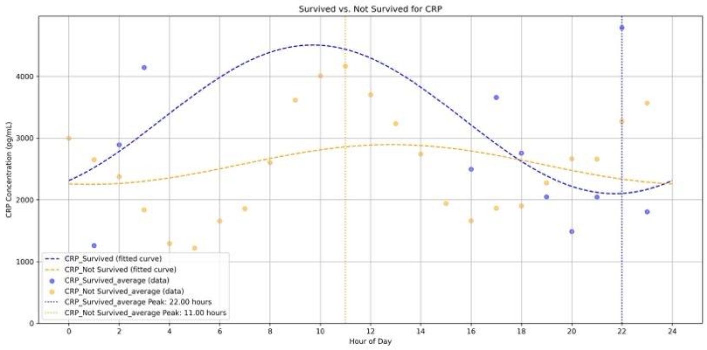

Metric	Value
Survived amplitude estimate	-1202.27
Not Survived amplitude estimate	-321.041
Amplitude difference estimate	-881.233
P-value for amplitude difference	0.92682
Survived peak time hours	22
Not Survived peak time hours	11
Phase difference estimate	3.141168
P-value for difference in phase	0.632249

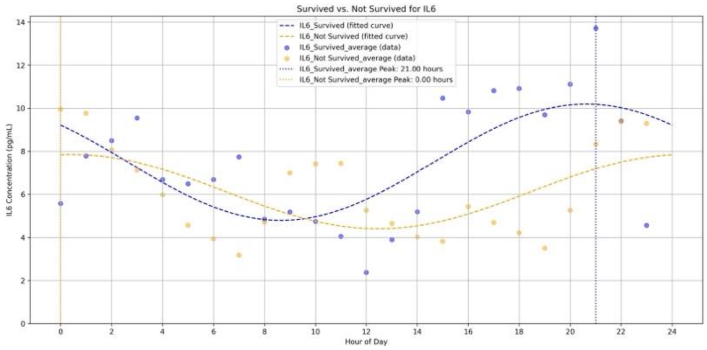

Metric	Value
Survived amplitude estimate	2.699316
Not Survived amplitude estimate	1.722852
Amplitude difference estimate	0.976464
P-value for amplitude difference	0.07081
Survived peak time hours	21
Not Survived peak time hours	0
Phase difference estimate	3.82765
P-value for difference in phase	0.648771

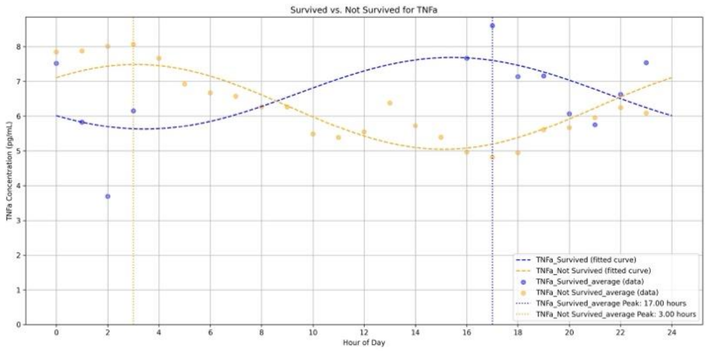

Metric	Value
Survived amplitude estimate	-1.03066
Not Survived amplitude estimate	1.220862
Amplitude difference estimate	-2.25152
P-value for amplitude difference	0.37706
Survived peak time hours	17
Not Survived peak time hours	3
Phase difference estimate	23.66855
P-value for difference in phase	0.260942
